# Draft genome assembly and sequencing dataset of the marine diatom *Skeletonema* cf. *costatum* RCC75

**DOI:** 10.1016/j.dib.2022.107931

**Published:** 2022-02-05

**Authors:** Maria Sorokina, Emanuel Barth, Mahnoor Zulfiqar, Michiel Kwantes, Georg Pohnert, Christoph Steinbeck

**Affiliations:** a Institute for Inorganic and Analytical Chemistry, Friedrich Schiller University, Lessingstrasse 8, Jena, Germany; b Bioinformatics Core Facility, Friedrich Schiller University, Leutragraben 1, Jena, Germany

**Keywords:** Genome sequencing, Diatoms, Bacillariophyceae, PacBio sequencing, Illumina sequencing, *Skeletonema costatum*, Algal genome

## Abstract

Diatoms (Bacillariophyceae) are a major constituent of the phytoplankton and have a universally recognized ecological importance. Between 1,000 and 1,300 diatom genera have been described in the literature, but only 10 nuclear genomes have been published and made available to the public up to date. *Skeletonema costatum* is a cosmopolitan marine diatom, principally occurring in coastal regions, and is one of the most abundant members of the *Skeletonema* genus. Here we present a draft assembly of the *Skeletonema cf. costatum* RCC75 genome, obtained from PacBio and Illumina NovaSeq data. This dataset will expand the knowledge of the Bacillariophyceae genetics and contribute to the global understanding of phytoplankton's physiological, ecological, and environmental functioning.

## Specifications Table

**Table:** 

Subject	Omics
Specific Subject Area	Genomics
Type of Data	Table, Raw data, genome sequences in Fasta format
How the data was acquired	Genome sequence was acquired using Pacbio Sequel I and Illumina NovaSeq PE150
Data Format	Raw, analysed and filtered data
Description of Data Collection	The strain RCC75 was grown in a seawater medium for 10 days. Later it was split into four samples which were used for DNA Extraction and sequencing.
Data Source Location	Institute: Roscoff Culture Collection Town: Roscoff Country: France
Data Accessibility	This Whole Genome Sequencing project has been deposited at DDBJ/ENA/GenBank under the accession number JAHBBA000000000. The version described in this paper is version JAHBBA010000000. The raw data is available on NCBI SRA with the accession number PRJNA647329 at https://www.ncbi.nlm.nih.gov/bioproject/647329.

## Value of the Data

• The Genome assembly data of *Skeletonema costatum* RCC75 is an addition to the only 10 published nuclear genomes from the Bacillariophyceae class.
• The algal research community will benefit from this data with its descriptive side of the species genome and how it relates to other *Skeletonema* sp.. It will allow exploring the similarities and differences between the different species within the *Skeletonema* genus, and the *Skeletonema costatum* species.
• This resource will improve the comprehension of metabolic pathways and lead to more marine natural products identification.

## 1 Data Description

Members of the Bacillariophyceae, commonly called diatoms, are unicellular siliceous algae of the complex phytoplankton community accounting for major primary production in aquatic ecosystems [1]. Diatoms have a large impact on marine silicon biogeochemical cycling as the gross production of biogenic silica exceeds the net oceanic floor silica deposition by a factor of 40 [2]. Because of their abundance and ability to fix carbon, they are also the major producers of oceanic, organic carbon and are hence large determinants of the global carbon cycle [3]. Currently, between 1,000 and 1,300 diatom genera are described, but only 10 nuclear genomes within the Bacillariophyceae have been published until now.

The genus *Skeletonema* comprises unicellular photosynthetic species with distinctive elliptical cells longitudinally stacked to form a colony of up to 24 cells [4]. The colony formation provides optimal survival in unstable and turbulent marine environments [5]. The cells within these chains (or colonies) are connected via long tubular projections called intercalary fultoportula processes (IFPPs). As with most diatoms, the cells take up silicic acid to produce biogenic silica that biomineralizes into a rigid silicified structure, known as frustule [6].

**Fig. 1:**
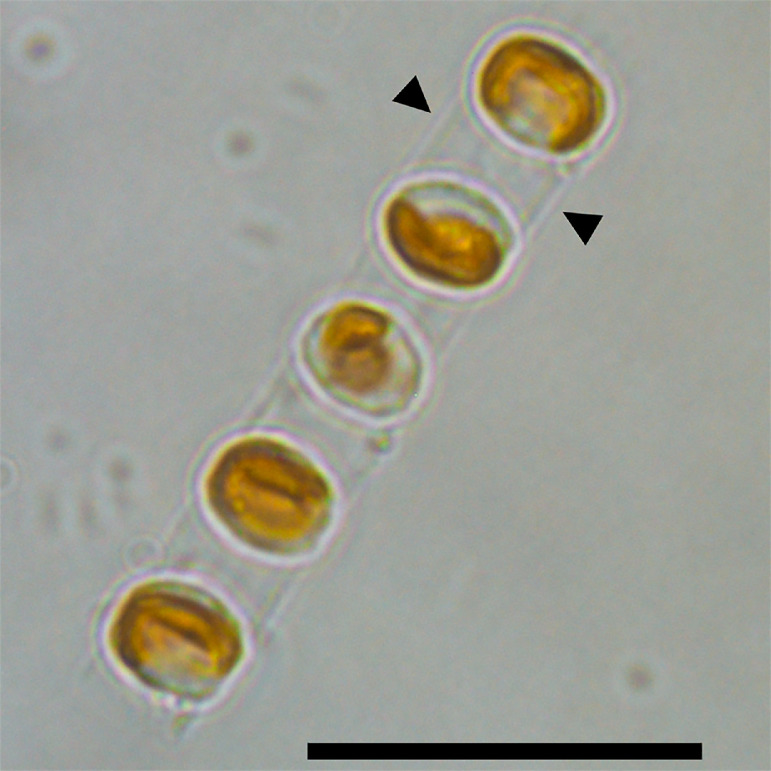
Bright-field light microscopy image of an *S. costatum* RCC75 filament consisting of five cells. For the upper pair of cells, the connecting processes are indicated by triangles. Scale bar, 20 µm.

*Skeletonema costatum* (Fig. 1) is one of the most cosmopolitan and abundant species of genus *Skeletonema* [7] and is principally distributed in the coastal regions [4]. Due to their genetic variability and ecological diversity, these diatoms are well adapted to different environmental conditions and levels of salinity [8]. They are also an excellent paleoenvironmental indicator [9]. *S. costatum* can form algal blooms under optimum conditions. These blooms lead to an increased phytoplankton concentration in the oceans and are promoted by environmental factors such as changes in nutritional content, temperature, and atmospheric deposition [10]. Previously, to discover putative genes associated with an algal bloom, Ogura *et al*. sequenced and described the genome of *S. costatum* [11] During the same study, a transcriptome analysis under varying light conditions, temperature, and nutrients was performed and described, and the RNA sequence data was released on DDBJ (DRA007346).

The presented genome assembly of *S. costatum* and the raw sequencing data are openly and freely available within the BioProject PRJNA647329 in open FASTA format.

## 2 Experimental Design, Materials and Methods

### 2.1 Sample culture and DNA extraction

Here, we report the genome sequence of *Skeletonema costatum* RCC75, which was obtained from the Roscoff Culture Collection (Roscoff, France). The strain was grown in F/2 medium under a 14/10 h light/dark regime with an illumination of 15–24 µmol photons m−2 s−1 for 10 days as standing cultures at 18°C, without additional nutrients supplementation. On day 10, the culture was dense enough to be clearly visible with the naked eye and was then harvested in four samples of 50mL using a needleless syringe. Each sample was then filtered with Durapore 5.0 µm filters, which eliminated most of the obligatory culture microbiome. The filters with diatom cells on them were then inserted in 2 mL microtubes without scraping off the cells. The microtubes were flash-frozen with liquid nitrogen and stored until DNA extraction at −80°C.

DNA was extracted from all four samples using the DNeasy® Plant Mini Kit (Qiagen). Silicon carbide beads (1 mm, BioSpec) were added to each Eppendorf Tube. The cells were then lysed by the 1 mm beads on a beating mill (Qiagen TissueLyser II, 3 × 1 min at frequency 30 Hz, with 1 min at room temperature between each run). The manufacturer's instructions were followed from there, with the exception of the final elution step where the provided elution solution was replaced by an EDTA-free one, following the recommendations of the sequencing facility. The genomic DNA concentration was determined with a Qubit 3.0 (Thermofisher) and a SpeedVac was used to concentrate the DNA. The DNA samples were then frozen at -80°C until the sequencing.

### 2.2 Genomic DNA sequencing

The genome sequencing was then performed by the commercial company Novogene (Cambridge, United Kingdom), using two parallel approaches, long reads with Pacbio Sequel I and a fine map with Illumina NovaSeq PE150.

According to the protocol provided by Novogene, the first step in the library construction for the Illumina fine-map sequencing and quality control consisted in the random fragmentation by sonication of the genomic DNA. The DNA fragments were then end-polished, A-tailed, and ligated with the full-length adapters of Illumina sequencing, and followed by further PCR amplification with P5 and indexed P7 oligos. The PCR products as the final construction of the libraries were purified with the AMPure XP system. Then libraries were checked for size distribution by Agilent 2100 Bioanalyzer (Agilent Technologies, CA, USA), and quantified by real-time PCR. The qualified libraries were then fed into Illumina sequencers, producing 2Gb of raw data.

For the PacBio sequencing, the first step in the generation of the SMRTbell library, required for this sequencing technology, was the generation of double-stranded 20k DNA fragments, by random DNA shearing. The SMRTbell library itself was produced by ligating universal hairpin adapters onto double-stranded DNA fragments. The hairpin dimers formed during this process were removed at the end of the protocol using a magnetic bead purification step with size-selective conditions. Adapter dimers were also removed using the PacBio MagBead kit. The final step of the library preparation protocol was to remove failed ligation products through the use of exonucleases. After the exonuclease and AMPure PB purification steps, the sequencing primer was annealed to the SMRTbell templates, followed by binding of the sequencing polymerase to the annealed templates. The sample was then sequenced on the PacBio Sequel platform, producing 25Gb of raw data.

### 2.3 Genome assembly

The genome assembly was performed by the Bioinformatics Core Facility Jena (BiC). The sequencing qualities of the PacBio long reads and the Illumina short reads were monitored using *LongQC* [12] (version 1.2.0) and *FastQC* [13] (version 0.11.9). Before assembly, all raw reads were checked for possible contamination with *Kraken 2* [14] (version 2.1.1). In addition to the standard *Kraken 2* libraries (archaea, bacteria, plasmid, viral, and human), we created and added three additional libraries based on the three available diatom genome assemblies of *Thalassiosira pseudonana* (GCF_000149415.2), *Thalassiosira oceanica* (GCA_000296205.1), and *Skeletonema costatum*[11] to provide a higher read classification resolution. Only reads that were classified as *T. pseudonana, T. oceanica, S. costatum*, or that could not be classified were kept for assembly. The genome assembly was performed with *Flye* [15] (version 2.8.1) using the parameters *–pacbio-raw* and *-g 30m.* For polishing the genome assembly, the filtered Illumina short reads were aligned to the draft assembly obtained from *Flye* using *Hisat2* [16] (version 2.2.1) with default parameters but not allowing reads to be spliced. Based on the short alignments, the genome assembly sequence was polished using *Pilon* [17] (version 1.23.2). A final assembly report was created utilizing *Quast* [18] (version 5.0.2), and the genome assembly statistics are shown in Table 1. Further re-sequencing will be needed to close the gaps in the draft genome sequence presented in this note and improve the overall genome quality.

**Table 1:** Genome assembly statistics from Quast analysis.

# contigs	1282
# contigs (> = 1,000 bp)	1,242
# contigs (> = 50,000 bp)	304
Total length	51,134,913
Total length (> = 1,000 bp)	51,104,503
Total length (>= 5000 bp)	50,448,718
Total length (>= 25000 bp)	43,834,615
Total length (>= 50000 bp)	36,634,768
Largest contig	756,974
N50	97,960
N75	42,259
L50	147
L75	342
GC (%)	45.13

**Mismatches**	
# N's	2,800
# N's per 100 kbp	5.48
Predicted genes	
# predicted genes (unique)	27,770
# predicted genes (>= 0 bp)	28,308 + 79 part
# predicted genes (>= 300 bp)	24,999 + 75 part
# predicted genes (>= 1500 bp)	7,002 + 18 part
# predicted genes (>= 3000 bp)	1,487 + 6 part

### 2.4 Code availability

The code containing the genome assembly workflow is available at Zotero [19].

## Ethics Statements

Not applicable.

## CRediT Author Statement

**Maria Sorokina:** Project Coordination, DNA extractions and writing the manuscript; **Emanuel Barth:** Genome Assembly; **Christoph Steinbeck:** Project supervision and obtaining the funds. **Georg Pohnert:** Project supervision and obtaining the funds, Samples provision; **Mahnoor Zulfiqar:** draft writing; **Michiel Kwantes:** DNA extractions. All authors reviewed the manuscript.

These authors contributed equally: Maria Sorokina, Emanuel Barth.

These authors jointly supervised this work: Christoph Steinbeck, Georg Pohnert.

## Declaration of Competing Interest

The authors declare that they have no known competing financial interests or personal relationships which have or could be perceived to have influenced the work reported in this article.
